# Atypical spatiotemporal activation of cerebellar lobules during emotional face processing in adolescents with autism

**DOI:** 10.1002/hbm.25349

**Published:** 2021-02-02

**Authors:** Charis Styliadis, Rachel Leung, Selin Özcan, Eric A. Moulton, Elizabeth Pang, Margot J. Taylor, Christos Papadelis

**Affiliations:** ^1^ Laboratory of Medical Physics, School of Medicine Aristotle University of Thessaloniki Thessaloniki Greece; ^2^ University of Toronto Toronto Canada; ^3^ Laboratory of Children's Brain Dynamics, Division of Newborn Medicine, Boston Children's Hospital Harvard Medical School Boston Massachusetts USA; ^4^ Center for Pain and the Brain, Department of Anesthesiology, Critical Care and Pain Medicine, Boston Children's Hospital Harvard Medical School Boston Massachusetts USA; ^5^ Department of Ophthalmology, Boston Children's Hospital Harvard Medical School Boston Massachusetts USA; ^6^ Division of Neurology Hospital for Sick Children Toronto Ontario Canada; ^7^ Neurosciences and Mental Health Program, Research Institute Hospital for Sick Children Toronto Canada; ^8^ Diagnostic Imaging Hospital for Sick Children Toronto Canada; ^9^ Autism Research Unit Hospital for Sick Children Toronto Canada; ^10^ Jane and John Justin Neurosciences Center Cook Children's Health Care System Fort Worth Texas USA; ^11^ Department of Bioengineering University of Texas at Arlington Arlington Texas USA; ^12^ Department of Pediatrics TCU and UNTHSC School of Medicine Fort Worth Texas USA

**Keywords:** adolescence, autism spectrum disorder, cerebellum, emotions, face processing, magnetoencephalography

## Abstract

Autism spectrum disorder (ASD) is characterized by social deficits and atypical facial processing of emotional expressions. The underlying neuropathology of these abnormalities is still unclear. Recent studies implicate cerebellum in emotional processing; other studies show cerebellar abnormalities in ASD. Here, we elucidate the spatiotemporal activation of cerebellar lobules in ASD during emotional processing of happy and angry faces in adolescents with ASD and typically developing (TD) controls. Using magnetoencephalography, we calculated dynamic statistical parametric maps across a period of 500 ms after emotional stimuli onset and determined differences between group activity to happy and angry emotions. Following happy face presentation, adolescents with ASD exhibited only left‐hemispheric cerebellar activation in a cluster extending from lobule VI to lobule V (compared to TD controls). Following angry face presentation, adolescents with ASD exhibited only midline cerebellar activation (posterior IX vermis). Our findings indicate an early (125–175 ms) overactivation in cerebellar activity only for happy faces and a later overactivation for both happy (250–450 ms) and angry (250–350 ms) faces in adolescents with ASD. The prioritized hemispheric activity (happy faces) could reflect the promotion of a more flexible and adaptive social behavior, while the latter midline activity (angry faces) may guide conforming behavior.

## Graphical abstract

The present study demonstrates distinct spatiotemporal patterns of cerebellar activity during happy and angry face processing in adolescents with autism spectrum disorder (ASD). ASD‐related emotional face processing during adolescence relates to a progression of overactivation of both hemispheric and midline cerebellar lobules. Our findings highlight that adolescents with autism spectrum disorder do not integrate cerebellar regions into the processing of emotional facial stimuli in a fashion similar to that of typically developing controls.

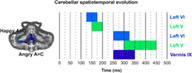

## 1 INTRODUCTION

Autism spectrum disorder (ASD) is a neurodevelopmental disorder of up to 2% prevalence. The pathogenesis of ASD is quite diverse (Chaste & Leboyer, 2012), and the consistent but heterogeneous dysfunction across brain systems indicates that there is little consensus on its neural basis (Frith, 2003; McPartland, Coffman, & Pelphrey, 2011). Abnormalities in the cerebellum, such as changes in cerebellar volume (Courchesne et al., 2001), vermal hypoplasia (Courchesne, Yeung‐Courchesne, Hesselink, & Jernigan, 1988; Hashimoto et al., 1995), reduced numbers of Purkinje and granule cells and changes in the inferior olive (Bauman & Kemper, 2005), are among the most consistent findings implicated in ASD (D'Mello, Crocetti, Mostofsky, & Stoodley, 2015; Sussman et al., 2015). Cerebellar lobules that consistently emerge as structurally abnormal in ASD are the Crus I, lobule VI, lobule VIII, hemispheric lobule IX, and vermal lobule IX (Cauda et al., 2011; Deramus & Kana, 2015; Stanfield et al., 2008; Stoodley, 2014; Yu, Cheung, Chua, & McAlonan, 2011). These lobules are associated with the severity of specific traits of ASD phenotype suggesting that the processing provided by cerebellum is relevant to a range of ASD symptoms (D'Mello et al., 2015; Rojas et al., 2006). Despite these solid and consistent pieces of evidence, the functional implications of the cerebellar anatomical abnormalities in ASD are still unclear.

The cerebellum, historically known for its role in motor control and coordination, is involved in a broad range of nonmotor functions (Buckner, 2013; Keren‐Happuch, Chen, Ho, & Desmond, 2014; Middleton & Strick, 1994; Schmahmann, 1991; Schmahmann & Sherman, 1998; Stoodley & Schmahmann, 2009; Strick, Dum, & Fiez, 2009), including emotion processing (Adamaszek et al., 2017; Baumann & Mattingley, 2012; Moulton et al., 2011; Styliadis, Ioannides, Bamidis, & Papadelis, 2015) and social cognition (Van Overwalle, Baetens, Mariën, & Vandekerckhove, 2014). Its involvement in emotional and social processing is anatomically supported via the cerebellar (Crus I/II, lobule IX, and posterior vermis in particular) interaction with regions of the cerebral cortex engaged in social processing and emotions (Kelly & Strick, 2003; Sokolov, Erb, Grodd, & Pavlova, 2014; Stoodley & Schmahmann, 2010). The cerebellum also plays a central role in higher‐order and nonmotor behaviors, including cognition and reward‐based learning. A recent animal study implicated the cerebellum in the primary circuits that regulate social behavior in mice such that interrupting the cerebellum's connection to the ventral tegmental area, the brain reward center, reduced social behavior (Carta, Chen, Schott, Dorizan, & Khodakhah, 2019). This finding may explain how the cerebellum contributes to the pathogenesis of diseases in which the dopaminergic system is dysregulated, such as in ASD (Carta et al., 2019).

Clinical evidence suggests that cerebellar lesions (VI, Crus I/II, and vermal IX regions in particular) can flatten or blunt emotions (Schmahmann, 2000; Schmahmann & Sherman, 1998; Schmahmann, Weilburg, & Sherman, 2007), thereby limiting one's capacity to communicate, empathize and bond with other people. Individuals with ASD similarly exhibit poor social functioning of varying severity and manifestation. When compared with typically developed (TD) individuals, adults with ASD demonstrated overactivation in functional magnetic resonance imaging (fMRI) of different neural structures, including the cerebellum, when perceiving neutral faces. This cerebellar overactivity was suggested to compensate for fusiform gyrus underactivation and therefore support face processing (Pierce, Müller, Ambrose, Allen, & Courchesne, 2001). Thus far, studies have not yet reached a consensus on cerebellum functional specificity or selectivity. Yet, there is a preference for negative emotional (angry and disgust) faces by hemispheric lobules VI, VIIA, and vermal VIII and IX as opposed to positive (happy and surprised) or neutral faces (Schraa‐Tam et al., 2012). The cerebellum indeed responds preferentially for emotional expressions, as patients with ischemic cerebellar lesions present significant impairments in the ability to match fearful faces to facial affect when compared with controls (Adamaszek et al., 2014). Similarly, cerebellar lesions can impair the recognition of fearful and angry facial features (Adamaszek et al., 2015), as well as specific social emotions (i.e., arrogance and guilt) (D'Agata et al., 2011).

Adults with ASD have been reported to show greater activation in the anterior vermis V during implicit face processing (happy and angry vs. neutral) compared to TD adults. Conversely, the latter showed greater midline activation in lobule V but only during explicit face processing (Critchley et al., 2000), which in turn suggests that vermis V may selectively engage in the processing of emotionally mixed valenced faces regardless of pathology (ASD/controls) or type of face processing (explicit/implicit). In contrast, there are reports that individuals with ASD exhibit cerebellar underactivation of hemispheric VI and Crus I/II during the implicit processing of emotional faces (Critchley et al., 2000; Deeley et al., 2007). Notably, overactivation, underactivation, or no differences in cerebral and cerebellar activity in persons with ASD (compared to controls) in response to facial expressions has been related to individual differences, such as symptom severity or task type (Nomi & Uddin, 2015; Scherf, Elbich, Minshew, & Behrmann, 2015). Yet, the available neuroimaging findings leave several unanswered questions. For instance, the particular emotional facial expressions that the cerebellum may preferentially process, the time course of these processes, and whether these processes unfold in a prioritized fashion in an emotionally developing population remain unclear.

The literature regarding cerebellar activations for adolescents with ASD is so far quite sparse. There are just a handful of fMRI studies examining cerebellar activations during implicit face processing on adults with ASD showing greater activation in vermis (Critchley et al., 2000) and underactivation of hemispheric lobules in participants with ASD compared to controls (Critchley et al., 2000; Deeley et al., 2007). Yet, ASD is a disorder that is typically diagnosed by 3 years of age and the affective facial processing regions develop dramatically between childhood and adulthood, especially during adolescence. Most pieces of evidence regarding cerebellar involvement in ASD were based on cerebellar metabolism and regional blood flow. This is possibly due to the fact that cerebellar electrophysiological responses were traditionally regarded as inaccessible to magnetoencephalography (MEG); although, new studies have challenged this notion (Andersen, Jerbi, & Dalal, 2020; Samuelsson, Sundaram, Khan, Sereno, & Hämäläinen, 2020; Styliadis et al., 2015). Contrary to fMRI which measures neural activity in an indirect way through hemodynamic responses, MEG is a neuroimaging technique that offers high levels of spatiotemporal precision; thus, it can provide a robust investigation of both the timing and location of brain activation at different frequency scales in response to emotional faces. MEG studies have reported beta band modulation during implicit emotion recognition of facial expressions when these became emotionally salient (Jabbi et al., 2015). This is particularly relevant for the study of ASD as another MEG study has shown reduced connectivity in the beta band in adolescents with ASD during implicit processing of angry faces (Leung, Ye, Wong, Taylor, & Doesburg, 2014). In this study, we aim to elucidate the spatiotemporal activation of cerebellar lobules in ASD during emotional face processing. We explored the time windows during which adolescents with ASD exhibited different emotion‐related cerebellar activities in comparison to TD controls. Based on previous sparse findings in the field, we hypothesize that vermis and the more posterior part of cerebellum, which show structural and functional alterations in ASD and are known to be active during implicit emotional processing (Siciliano & Clausi, 2020), will display differences in response to affective faces (compared to TD controls). Specifically, we hypothesize that adolescents with ASD would exhibit a vermal overactivation and a hemispheric underactivation in line with previous findings (Critchley et al., 2000; Deeley et al., 2007) in the beta frequency band. To test our hypothesis, we recorded evoked responses from adolescents with ASD and TD controls using MEG during implicit emotional face processing of happy and angry faces. So far, MEG investigations in individuals with ASD have studied exclusively the cerebral activity during emotional face processing (Bailey, Braeutigam, Jousmäki, & Swithenby, 2005; Kovarski et al., 2019; Leung et al., 2015; Leung, Pang, Anagnostou, & Taylor, 2018; Leung, Pang, Brian, & Taylor, 2019; Safar et al., 2020; Wright et al., 2012). Tracking the time windows in the millisecond range can provide insights on how the cerebellar activations in response to affective faces compare with the well‐known cerebral mediators (i.e., insula, fusiform) of face processing, at well‐studied latencies (i.e., N170, Bentin, Allison, Puce, Perez, & McCarthy, 1996). This approach can be paramount in understanding the complexities of cerebellar dysfunction in ASD‐related face processing.

## 2 MATERIALS AND METHODS

### 2.1 Participants

The study included 24 adolescents (age range = 12–15 years) diagnosed with ASD (20 males, 14.03 ± 1.20 years, 23 right‐handed, 7 medicated, IQ = 90.79 ± 23.76), and 24 healthy controls (19 males, 14.27 ± 1.12 years, 23 right‐handed, IQ = 110.04 ± 12.21). The diagnosis of ASD was informed either by module 3 or module 4 of either the Autism Diagnostic Observation Schedule‐Generic (ADOS‐G) (Lord et al., 2000) or the Autism Diagnostic Observation Schedule‐2 (ADOS‐2) (Lord et al., 2012) and confirmed by expert clinical judgment. The mean and *SD* of total scores (ADOS and ADOS‐2 pooled) were 11.30 ± 3.30, which was well above the clinical threshold. IQ estimation was based on two subtests (vocabulary and matrix reasoning) of the Wechsler Abbreviated Scale of Intelligence. Exclusion criteria for both groups were as follows: (a) history of neurological or neurodevelopment disorders (other than ASD for participants in the clinical group), (b) acquired brain injury, (c) impaired visual acuity, (d) color blindness, (e) IQ≤65, and (f) standard contraindications to MRI and MEG acquisition. The use of psychotropic medications was an exclusion criterion for the TD group. Seven of our participants with ASD were on medication (i.e., Bicentin, Celexa, Cipralex, Concerta, Gabapentin, Ritalin, Seroquel, Strattera, and Zeldox). The Hospital for Sick Children Research Ethics Board approved the study. The participants and their legal guardians gave their written informed consent in compliance with the Code of Ethics of the World Medical Association (Declaration of Helsinki) and the standards established by the Hospital for Sick Children Research Ethics Board.

### 2.2 Emotional face stimuli

The stimuli were 25 different faces (13 males, 12 females) for each of three expressions (i.e., happy, angry, and neutral) from the NimStim Set of Facial Expressions (Tottenham et al., 2009), as described by Leung et al. (2015). The criteria in selecting happy and angry facial expressions were to choose only those faces categorized correctly at a minimum of 80% accuracy (Tottenham et al., 2009). Each of the selected faces was divided into 64 cells and randomized to create unique scrambled patterns corresponding to each face. A mosaic was applied to the image (15 cells per square), after which a Gaussian blur was applied (10.0°) using Adobe Photoshop. Pairs of faces and scrambled patterns were matched for luminosity and color.

### 2.3 Implicit processing of happy and angry faces in ASD

The experimental design was implemented using specific elements that aided in achieving consistent responses during the recordings. Individuals with ASD exhibit impairments for both implicit and explicit components (Lozier, Vanmeter, & Marsh, 2014; Senju, 2013). The use of implicit emotion processing was based on the fact that: (a) there are more convergent findings regarding the existence of implicit emotional processing impairment in ASD (Baron‐Cohen, Jolliffe, Mortimore, & Robertson, 1997) but controversial ones for the explicit elaboration of emotions (see Siciliano & Clausi, 2020 for a discussion on this issue); (b) individuals with ASD have greater difficulty with explicitly recognizing affect relative to controls, and thus using an explicit emotional face processing task would have resulted in different levels of performance between the two groups; and (c) an implicit task enhances the ecological validity of the study given that adaptive social behavior necessitates automatic and rapid emotional processing and produces stronger limbic (i.e., amygdala and anterior cingulate) (Critchley et al., 2005) and cerebellar responses in individuals with ASD (Critchley et al., 2000). We chose to present happy and angry facial expressions because the former serves as a definite positive display given that happiness is a prevalent emotion and youth with ASD do not derive the same social reward from happy faces as do their TD peers (Sepeta et al., 2012). In turn, anger, rather than fear was chosen as a definite negative display given that the processing of anger appears to involve a greater understanding of social norms and differs from fear in its communicative intent and social function (Frijda & Tcherkassof, 1997; Marsh, Ambady, & Kleck, 2005). Anger, while still considered a basic emotion, is usually exhibited in response to transgressions by another individual and both children with ASD and, to a greater extent, TD children can identify social reasons for anger (Rieffe, Meerum Terwogt, & Kotronopoulou, 2007). As such, it was not unexpected that individuals with ASD would have shown difficulty in processing angry faces (Kuusikko et al., 2009; Pelphrey et al., 2002), further confirming anger as an emotion of interest when investigating facial affect processing in ASD.

### 2.4 Experimental procedure

**FIGURE 1:**
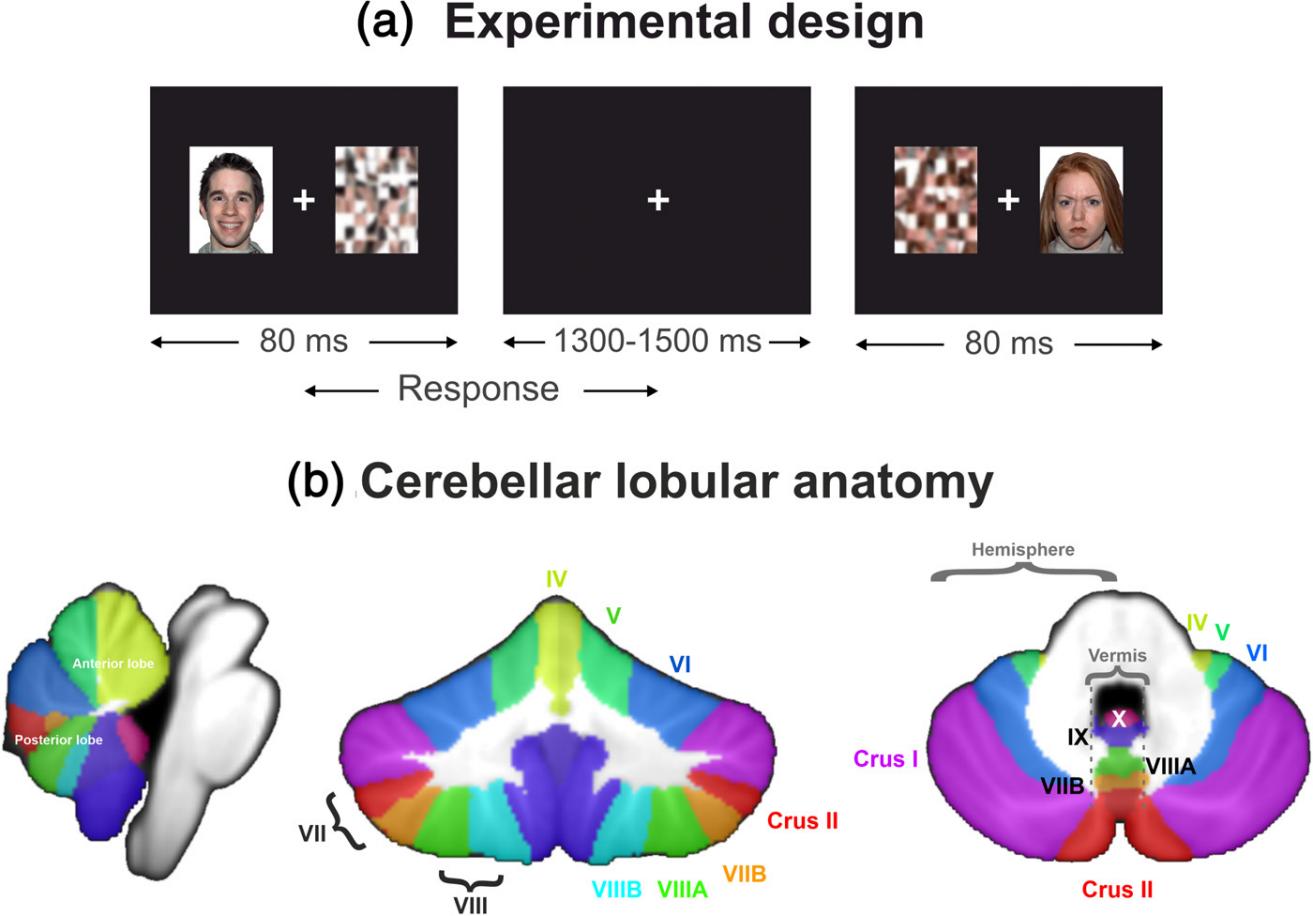
(a) Implicit emotional face processing task. Happy, angry, or neutral faces located in either left or right hemifield were presented concurrently with a scrambled pattern (target) in the other hemifield with a fixation cross in the center. Participants were instructed to press a button corresponding to the left or right position of the target. (b) Cerebellar lobular anatomy. Cerebellar anatomy is shown on coronal, sagittal, and axial slices through the Spatially Unbiased Infratentorial (SUIT) atlas (Diedrichsen, Balsters, Flavell, Cussans, & Ramnani, 2009). Anatomically (Schmahmann et al., 1999), the human cerebellum consists of the vermis and two hemispheres and is subdivided into three lobes (anterior, posterior, and flocculonodular and 10 lobules (I–X). Anterior lobules: I–V. Superior posterior lobules: VI, VII (subdivided into Crus I [VIIAf], Crus II [VIIAt], and VIIB. Inferior posterior and flocculonodular lobules: VIII (subdivided into VIIIA, VIIIB), IX, X. Yellow, lobules I–IV; light green, lobule V; blue, lobule VI; purple, lobule VII (Crus I); red, lobule VII (Crus II); orange, lobule VII (VIIB); green, lobule VIIIA; aqua, lobule VIIIB; dark purple, lobule IX; pink‐purple, X. The labeling of the cerebellar lobules is based on the nomenclature of Schmahmann, Doyon, and Toga (2000). The colormap of the lobules is based on the LUT table provided with SUIT (Diedrichsen et al., 2009)

The task stimuli (happy, angry, or neutral faces) were presented concurrently with a scrambled pattern, each on either side of a central fixation cross (Figure 1a). The scrambled faces provided well‐characterized nonaffective images, that were, matched by luminosity and color, to be presented concurrently with the emotional face. The task included 300 trials in total shown in randomized order. In particular, each face was shown twice in each hemifield: 50 trials of each of the three expressions were presented in the left and right hemifields. Left/right presentation of the emotional faces was counterbalanced across trials, and we did not consider the emergence of differences in lateralization of the face presentation (see Section 4.5 for a rationale). Presentation software (Neurobehavioral Systems, Inc., http://www.neurobs.com) controlled the stimuli presentation. Emotions were irrelevant to the task (implicit face processing). Participants were instructed to fixate on the central cross and press the left or right button on a response button box with respect to the scrambled pattern's side. The central fixation cross was used as a central point of interest while the target (scrambled pattern) and emotional face were presented bilaterally within the parafoveal region for implicit affect processing of the faces. As such, the central fixation cross served as a point of interest to discourage saccades and enhance attendance to the task at hand. Stimuli were projected for 80 ms with a varying interstimulus interval of 1,300–1,500 ms to minimize any saccades during the trials. This extremely rapid presentation of stimuli does not allow for any scanning of the presented images; therefore, differences in eye fixation did not play a role in differential activation that we observed between participants with ASD and controls. The images were back‐projected through a set of mirrors onto a screen, and the projections were delivered at a viewing distance of 79 cm from the participants' eyes. The visual angle of the stimuli was 6.9° and fell within the parafoveal region of view. Participants were instructed to press the button on a response button box that corresponded to the same side as a scrambled pattern while fixating on a central fixation cross. The button press in response to the scrambled pattern ensured that participants remained vigilant and were attending to the presentation of stimuli, and indicated whether there were any behavioral differences in emotional processing. Asking participants to attend to the scrambled pattern rather than the affective face, although both fell within the parafoveal region, allowed the study of implicit, rather than explicit, affective processing for reasons discussed above. All participants initially underwent a practice session outside the MEG to become familiar with and ensure understanding of the experimental task. A separate set of practice stimuli, that is, 10 trials, was used. The length of the practice session was consistent across groups/subjects. Then, they performed the aforementioned procedure in the MEG.

### 2.5 Data acquisition

MEG data were recorded at a sampling rate of 600 Hz using a 151‐channel CTF MEG system (MISL, Coquitlam, BC, Canada) with a bandpass of 0–150 Hz in a magnetically shielded room at the Hospital for Sick Children. The CTF MEG system features a third order spatial gradient to improve signal quality. All participants completed the experimental paradigm in a supine position with their head in the MEG dewar. Fiducial coils were placed on the left and right preauricular points and the nasion to monitor head position and movement within the dewar. Radio‐opaque markers for MRI coregistration replaced the fiducial coils. A T1‐weighted MR image (3D SAG MPRAGE: PAT, GRAPPA = 2, TR/TE/FA = 2,300 ms/2.96 ms/90°, FOV = 28.8 × 19.2 cm, 256 × 256 matrix, 192 slices, slice thickness = 1.0 mm isotropic voxels) was obtained for each participant with a 3 T MR scanner (MAGNETOM TimTrio, Siemens AG, Erlangen, Germany).

### 2.6 Behavioral analyses

Group effects in IQ, response accuracy, and response latencies across emotions were assessed using SPSS 25.0 software (SPSS Inc., Chicago, IL). ANCOVAs were conducted to examine group (ASD vs. control) and emotion (angry vs. happy) effects, with IQ as a covariate. Due to violation of ANCOVA assumptions, we transformed the accuracy data using the Logit transformation ln(*p*/(1‐*p*)).

### 2.7 Data preprocessing

We preprocessed the MEG data in Brainstorm (Tadel, Baillet, Mosher, Pantazis, & Leahy, 2011). We removed the DC offset, and MEG data were band‐pass filtered between 1 and 100 Hz with a notch filter applied at 60 Hz. The filtered signal was segmented into trials, each one lasting 650 ms, beginning 150 ms before and ending 500 ms after the onset of each trial. The epochs were time‐locked to the onset of each trial and averaged for each of the three emotional expressions across the participants. We manually excluded trials with head movement exceeding 10 mm (relative to median head position) using software developed in‐house. There were no significant differences in head movement between groups nor between the number of usable trials per condition between groups. This indicates similar levels of signal‐to‐noise ratio for ASD and TD participants per condition (total number of usable trials for ASD per condition: 1,900 for happy, 1,900 trials for angry, 1,900 for neutral; and for TD per condition: 2,298 for happy, 2,300 trials for angry, 2,298 for neutral). We removed artifactual signal components in MEG data (i.e., eye blinks and cardiac artifacts) with the use of the independent component analysis (ICA) as provided by Brainstorm (Tadel et al., 2011).

### 2.8 MEG sensor space time–frequency analysis

Time–frequency (TF) decomposition of MEG oscillatory activity was quantified by continuous Morlet wavelet transformation using Brainstorm (Tadel et al., 2011). Epochs were from −150 to 500 ms relative to the onset of the angry and happy stimuli. The analysis was performed only for participants whose MEG sensors were covering the entire cerebellum during the experiment. We calculated the average of the TF maps for each trial, sensor, and participant across the frequency range of 1–100 Hz to provide an analysis of phase‐locked information. Then, we standardized the TF values [event‐related perturbation; event related synchronization/desynchronization (ERS/ERD)]. We performed power F‐tests against baseline (from −150 to 0 ms) for within‐group statistical analysis of the TF representations of power for both happy and angry facial stimuli conditions as well as permutation *t* tests (equal variance, Monte‐Carlo, 10,000 randomizations) for between‐group statistical analysis of the TF representations of power for the happy and the angry facial stimuli conditions. We chose a Type‐I error rate of *α* = 5% with correction from multiple comparisons by adjustment of the false discovery rate (FDR). We controlled multiple comparisons for all dimensions (i.e., signals, time, and frequency) and thus all values in the dataset were considered as the same repeated test, and only one corrected *p*‐threshold was computed for all the time samples and all sensors. We considered the effects significant at *p* < .05 (FDR level correction for multiple comparisons). Details on Brainstorm's implementation for FDR control, which is the Benjamini–Hochberg step‐up procedure, can be found elsewhere (Benjamini & Hochberg, 1995). In short, the Benjamini–Hochberg step‐up procedure is the following: (a) sort the *p* values *p*(*k*) obtained across all the multiple tests (*k* = 1…Ntest), (b) find the largest *k* such as *p*(*k*) < *k*/Ntest × *α*, and (c) reject the null hypotheses corresponding to the first *k* smallest *p* values.

### 2.9 MEG source analysis

We computed a volume head model for each subject based on the merged surfaces of the cortex and cerebellum. The cortex was generated from individual T1's using the SPM12 canonical surfaces option within Brainstorm, while the cerebellum's segmentation was imported from Freesurfer (Fischl, 2012). We used the overlapping‐spheres approach to obtain a MEG forward model (Huang, Mosher, & Leahy, 1999) that seems to have comparable accuracy to the boundary element method. The overlapping spheres method is based on the estimation of a different sphere for each sensor. We checked that the spheres and source grid covered the volume of interest (i.e., cortex and cerebellum). We estimated the noise covariance from the MEG recordings for a time window from −150 to 0 ms. We used dynamic statistical parametric mapping (dSPM) (Dale et al., 2000) for source estimation on the neural responses of each participant for each emotion at 1–30 Hz, as provided by Brainstorm (Tadel et al., 2011) using parameters that produce unconstrained solutions. In conjunction with the TF results at the sensor level which indicated significant differences within and between the two groups for the emotional faces in frequencies ~5 and 30 Hz (see Section 3.2), we restricted our source analysis in the frequency band of 1–30 Hz in order to enhance potential differences between the two groups in this frequency band at the source level. dSPM is a depth‐weighted minimum norm (Dale et al., 2000) based on the whitened and depth‐weighted linear L2‐minimum norm (wMNE) estimates algorithm. dSPM assumes that extended sources over the brain volume can generate the observed MEG activity. To assess the cerebellar spatiotemporal evolution of emotional face processing, we used a sliding window approach from 50 to 500 ms, using sliding time windows of 50 ms in duration, overlapping by 25 ms (i.e., 50–100, 75–125 ms, 100–150 ms … 400–450 ms, 425–475 ms, 450–500 ms) for a total of 17 time windows.

### 2.10 Group analysis of MEG source activity

We exported the volumetric images for each emotional expression condition from Brainstorm and imported these to SPM12 (https://www.fil.ion.ucl.ac.uk/spm) for second‐level analysis. We resliced the volumetric images to 2 × 2 × 2 mm per voxel resolution using the “Coregister” module of SPM12. Neutral faces were included to be used as an emotional baseline. Given the ambiguity of neutral faces, these are often misinterpreted, particularly by children (Thomas et al., 2001), and may not truly represent an emotionally neutral baseline in ASD (Dawson, Webb, Carver, Panagiotides, & McPartland, 2004; Eack, Mazefsky, & Minshew, 2015). For instance, children with ASD have been shown to fail in distinguishing between neutral and fearful faces (Dawson et al., 2004), while adults with ASD can misinterpret and attribute negative valence to neutral faces (Eack et al., 2015). Thus, neutral faces were not included in our statistical design similar to the previous work of our group (Leung et al., 2019, 2015). We used Sandwich Estimator (SwE) Toolbox (http://www.nisox.org/Software/SwE/) (Guillaume et al., 2014) to perform the group‐level analysis via a two‐sample unpaired test within a gray‐matter mask of the cerebellum. The cerebellar responses of the ASD group to happy faces were contrasted to those of the TD group. Similarly, the cerebellar responses of the ASD group to angry faces were contrasted to those of the TD group. As a mask, we used the Spatially Unbiased Infratentorial (SUIT) atlas (http://www.diedrichsenlab.org/imaging/suit.htm) (Diedrichsen et al., 2009), which was also resliced using the “Coregister” module of SPM12. We used Wild Bootstrap (WB) to estimate nonparametric inferences for the defined contrasts, and calculated familywise error (FWE) corrected *p* values at a voxel‐cluster level via the threshold free cluster enhancement (TFCE) approach (Smith & Nichols, 2009). This approach ensures proper control of FWE and provides better sensitivity than other methods over a wide range of test signal shapes and signal‐to‐noise‐ratio values. The choice of TFCE inference allows for the calculation of voxelwise and TFCE FWE *p* values, and TFCE uncorrected *p* values using WB. All second‐level contrasts were corrected for multiple comparisons at the TFCE level with an alpha of .05.

### 2.11 Cerebellar anatomy and probabilistic maps

We identified the regions of significance using the probabilistic atlas of the cerebellar lobules (Diedrichsen et al., 2009), which are available through the Anatomy toolbox (Eickhoff et al., 2005). We characterized significant activations with the use of the assignment algorithm (Eickhoff, Heim, Zilles, & Amunts, 2006), which bases the assignment of each voxel to the most probable anatomical area at the position under investigation. We set the probability limit for the assignment to ≥80%, and in the case that the activity could be assigned in two distinct cerebellar lobules, we chose the region with a higher probability. We have previously used these tools to identify the cerebellar lobules involved in emotional processing for healthy adults (Styliadis et al., 2015). As in our previous study, we labeled the cerebellar lobules using the nomenclature of Schmahmann et al. (2000). Figure 1b shows the cerebellar lobular anatomy.

## 3 RESULTS

### 3.1 Study cohort

We analyzed MEG data from 19 adolescents (age range = 12–15 years) diagnosed with ASD (16 males, 14.02 ± 1.22 years, 17 right‐handed) and 23 TD controls (18 males, 14.25 ± 0.99 years, 22 right‐handed) for whom MEG sensors were well‐covering the entire structure of cerebellum. Supplementary Table 1 lists the labels and the number of MEG sensors covering the cerebellum for each participant. The IQ was significantly lower in adolescents with ASD (97.58 ± 11.89) than in controls (111.65 ± 12.07), *t*(40) = 3.798 *p* = .001. A 2 (emotion: happy, angry) × 2 (group: ASD, controls) ANCOVA with IQ as a covariate showed a between‐group effect on accuracy, *F*(1, 76) = 6.08, *p* = .016. Adolescents with ASD (0.853 ± 0.15) were significantly less accurate than the controls (0.95 ± 0.05). Follow‐up ANCOVAs revealed no significant effects or interactions. A 2 (emotion: happy, angry) × 2 (group: ASD, controls) ANCOVA with IQ as a covariate showed no main or interaction effects on response latency.

### 3.2 TF analysis

**FIGURE 2:**
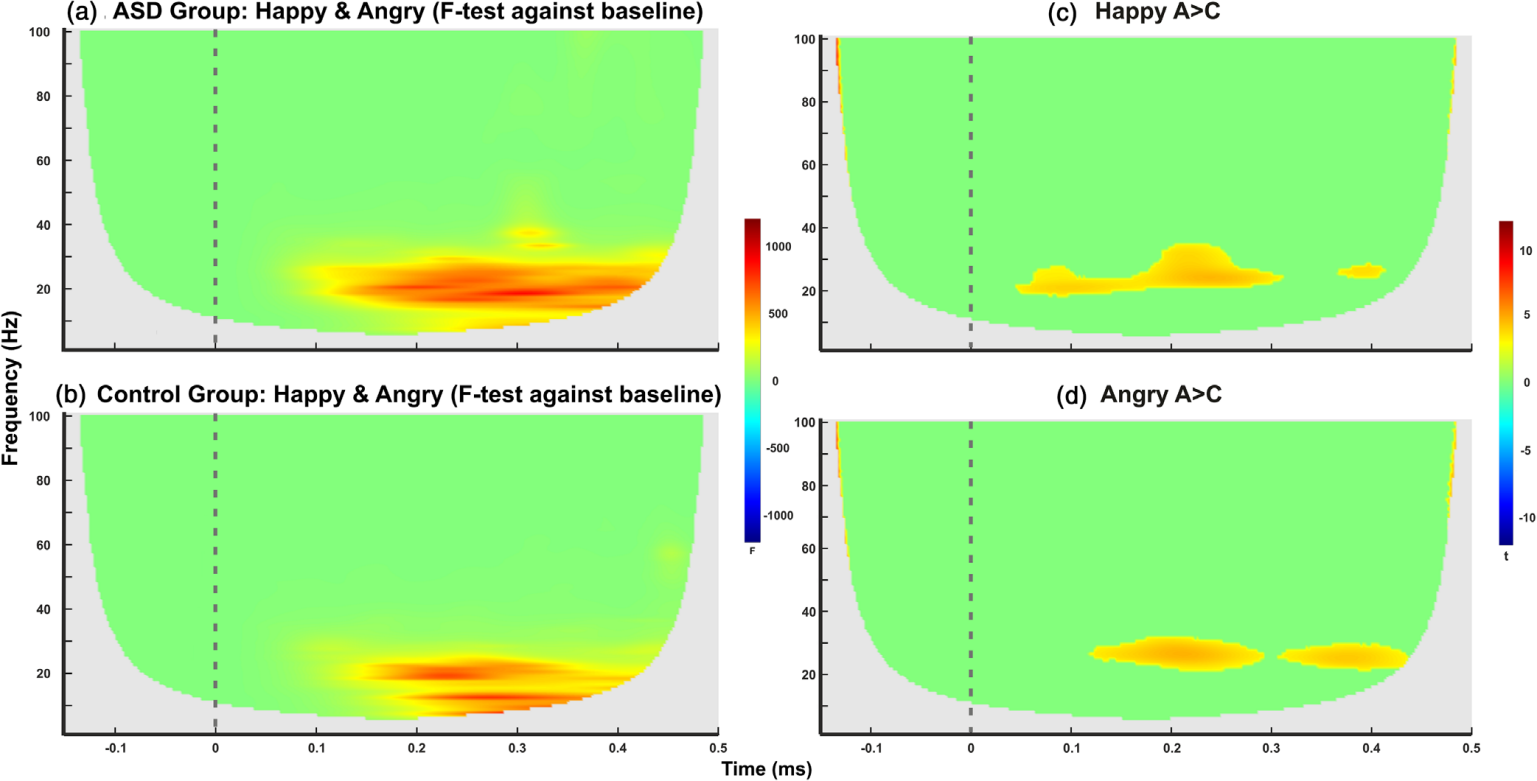
Time–frequency plots for a representative set of magnetoencephalography (MEG) sensors that cover the cerebellum showing (i) significant within‐group differences at *p <* .05 (false discovery rate [FDR] correction for multiple comparisons) for both happy and angry faces for (a) autism spectrum disorder (ASD) and (b) typically developing (TD) group, and (ii) significant between‐group differences at *p <* .05 (FDR correction for multiple comparisons) for (c) happy and (d) angry faces for the power estimated from the output of the Morlet wavelet transform from 1 to 100 Hz (y‐axis) over time from −150 to 500 ms (x‐axis) relative to the stimulus onset. Edge effects were cut out from the time–frequency (TF) maps after computation. Stimulus onset is at 0 ms. “A > C” denotes greater activation in the ASD group

Significant TF within‐group differences for both happy and angry facial stimuli for ASD (Figure 2a) and the TD group (Figure 2b) as well as between‐group differences for happy (Figure 2c) and angry (Figure 2d) faces are reported at *p <* .05, FDR corrected for multiple comparisons. Periods of significant TF ASD‐group differences for both happy and angry faces occur at ~80 ms and last till ~450 ms within 6–40 Hz. Periods of significant TF TD‐group differences for both happy and angry faces occur at ~100 ms and last till ~430 ms within 7–31 Hz. Periods of significant TF between‐group differences for happy faces occur at ~45 ms and last till ~315 ms for 17–35 Hz and then at ~365 ms till ~415 ms for 23–29 Hz, while for angry faces occur at ~120 ms and last till ~295 ms for 20–32 Hz and at 305 till 435 ms for 20–30 Hz.

### 3.3 Time course and source localization of cerebellar activity

**FIGURE 3:**
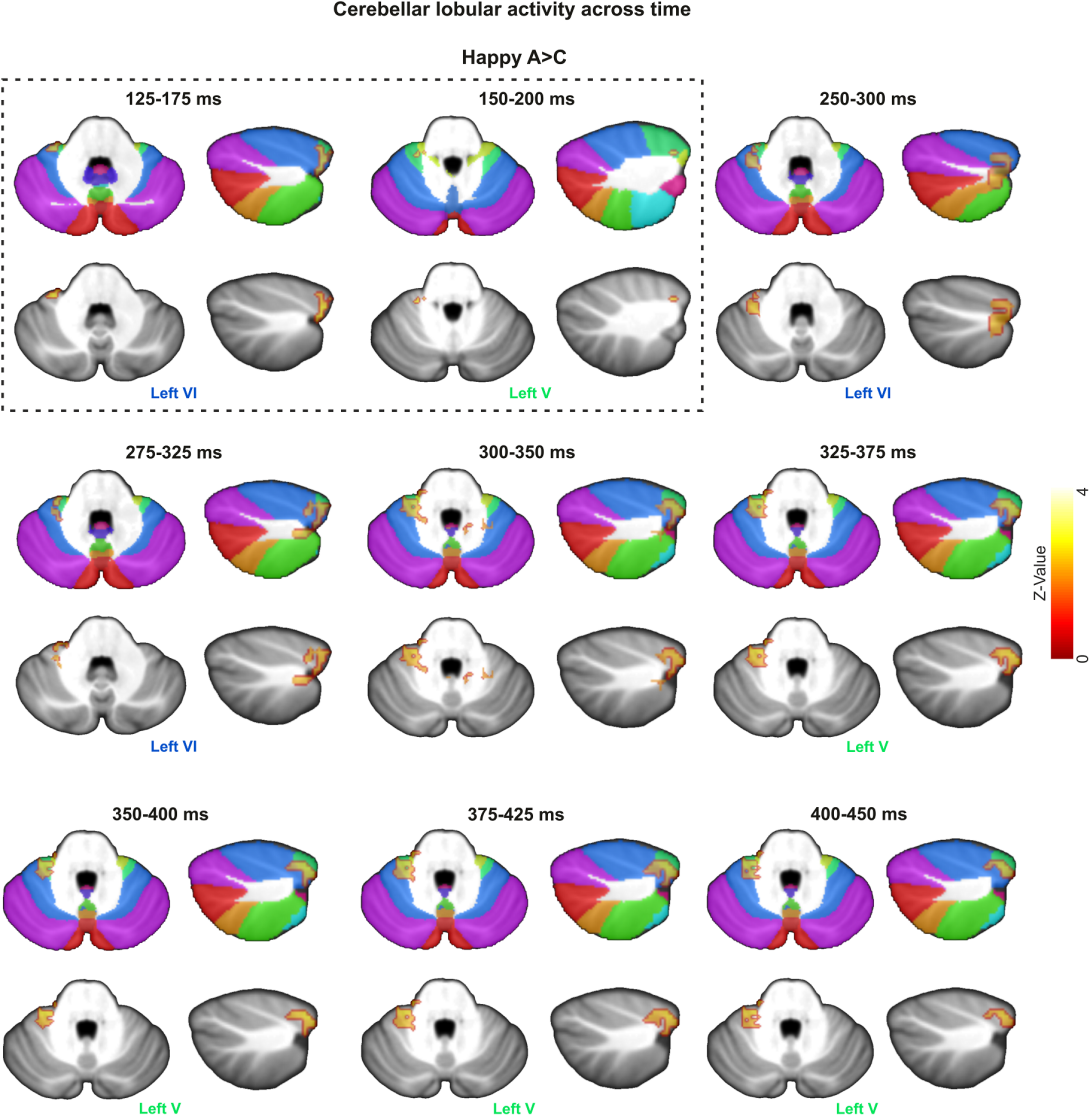
Source localization of significant between‐group differences for happy faces. Red to yellow color scale. The threshold is reported at *p <* .05 pFWE‐corr (threshold free cluster enhancement [TFCE]‐level). The data were visualized using MRIcron (https://people.cas.sc.edu/rorden/mricron/install.html) with Sandwich Estimator (SwE) maps as the overlay, and the Spatially Unbiased Infratentorial (SUIT) (Diedrichsen et al., 2009) template as the underlay. Happy effects are analyzed via one‐sided tests and therefore generate a Z value. Cerebellar lobules for happy faces are shown on axial and sagittal slices. The dotted rectangular contains the time windows associated with M170/N170

**FIGURE 4:**
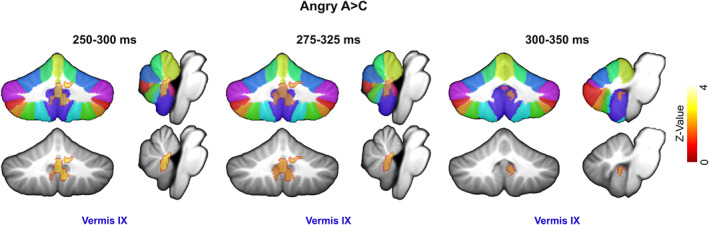
Source localization of significant between‐group differences for angry faces. Red to yellow color scale. The threshold is reported at *p <* .05 pFWE‐corr (threshold free cluster enhancement [TFCE]‐level). The data were visualized using MRIcron (https://people.cas.sc.edu/rorden/mricron/install.html) with Sandwich Estimator (SwE) maps as the overlay, and the Spatially Unbiased Infratentorial (SUIT) (Diedrichsen et al., 2009) template as the underlay. Angry effects are analyzed via one‐sided tests and therefore generate a Z value. Cerebellar lobules for angry faces are shown on coronal and sagittal slices

**FIGURE 5:**
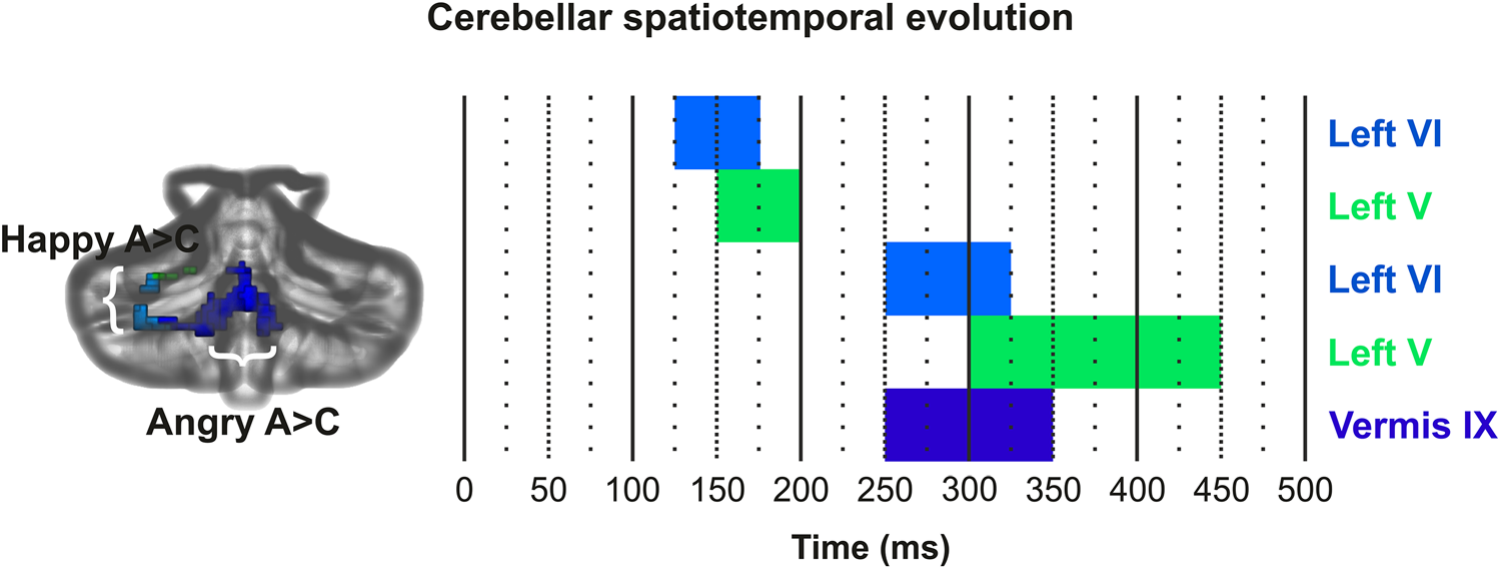
Temporal evolution of cerebellar emotional responses across time for a period of 500 ms. “A > C” denotes greater activation in the autism spectrum disorder (ASD) group. The data were visualized using MRIcroGL (https://www.mccauslandcenter.sc.edu/mricrogl/home) with Sandwich Estimator (SwE) maps as the overlay, and the Spatially Unbiased Infratentorial (SUIT) (Diedrichsen et al., 2009) template as the underlay

Adolescents in the ASD group exhibited stronger cerebellar activity in comparison to their TD counterparts for both happy (Figure 3) and angry faces (Figure 4). Figure 5 presents the temporal evolution of cerebellar emotional responses across time for a period of 500 ms. Table 1 lists the anatomical location (MNI coordinates) of the maxima of significant differences between ASD and TD groups for happy and angry faces, their corresponding statistical value at *p <* .05 pFWE (*p* value FWE rate)‐corr (TFCE‐level) and their cluster size (number of voxels) along the significant time course.

**TABLE 1:** Peak activations at *p <* .05 FWE‐corrected (TFCE‐level) for ASD adolescents and TD controls in response to (a) happy and (b) angry faces. “A > C” denotes greater activation in the ASD group. Results are superimposed on standardized MNI coordinates; vermis: −10 mm ≤ *x* ≤ +10 mm; left and right paravermal region: −24 mm ≤ x < −10 mm, +10 mm < x ≤ +24 mm; left and right lateral hemispheres: x < −24 mm, x > +24 mm; Z, z‐values for each peak; SwE toolbox gives z‐statistics in place of *t*‐statistics for pairwise contrasts; CS, cluster size in number of activated voxels along the significant time course

Time	Directionality	Laterality	Label	MNI (mm)			Z	CS	Puncor	TFCE‐level pFWE‐corr
				x	y	z				
*Happy*										
125–175	A > C	Left	VI	−30	−36	−34	2.84	94	0.002	0.031
150–200	A > C	Left	V	−22	−38	−28	2.51	36	0.008	0.047
250–300	A > C	Left	VI	−34	−40	−32	2.85	516	0.002	0.007
275–325	A > C	Left	VI	−30	−40	−30	2.64	643	0.004	0.012
300–350	A > C	Left	V	−28	−38	−30	2.84	272	0.003	0.020
325–375	A > C	Left	V	−28	−38	−30	2.95	164	0.002	0.008
350–400	A > C	Left	V	−28	−38	−30	2.87	123	0.002	0.007
375–425	A > C	Left	V	−28	−38	−30	3.11	211	0.001	0.011
400–450	A > C	Left	V	−28	−38	−30	2.80	162	0.003	0.019
*Angry*										
250–300	A > C	Right	Vermis IX	2	−56	−40	2.71	832	0.003	0.015
275–325	A > C	Right	Vermis IX	2	−56	−36	3.05	936	0.001	0.004
300–350	A > C	Right	Vermis IΧ	8	−52	−38	3.37	308	0.000	0.012

Abbreviations: ASD, autism spectrum disorder; FWE, familywise error; SwE, Sandwich Estimator; TD, typically developing; TFCE, threshold free cluster enhancement.

#### 3.3.1 Happy

Adolescents in the ASD group, in comparison to their TD counterparts, exhibited significantly stronger cerebellar activations in response to happy faces initially for a short period of 125–200 ms and then for a longer period of 250–450 ms (Figure 3). ASD‐related cerebellar responses to happy faces were localized to a left hemispheric cluster extending from lobule VI to lobule V. Specifically, adolescents with ASD initially exhibited an early and stronger cerebellar activity in left‐hemispheric lobules VI and V at 125–175 ms and 150–200 ms, respectively, and then at 250–325 ms following the presentation of happy facial stimuli (Figure 3). During the period between 325 and 450 ms, the maxima of cerebellar activity in response to happy facial stimuli shifted from left VI to left hemispheric lobule V (Figure 3).

#### 3.3.2 Angry

Adolescents in the ASD group, compared to TD controls, exhibited stronger cerebellar activations in response to angry faces for a period of 250–350 ms. ASD‐related cerebellar responses to angry faces were localized to midline cerebellum, in particular to vermal IX at 250–350 ms (Figure 4).

## 4 DISCUSSION

We mapped the evolution of spatiotemporal cerebellar activity during implicit (angry and happy) face processing for ASD and control populations by examining the modulations of MEG activity. The current MEG study is the first to address abnormal activity in the cerebellar lobules of adolescents with ASD during implicit emotional processing. We found that adolescents with a diagnosis of ASD, unlike their TD counterparts, exhibit: (a) more activation on left VI and V lobule for the prioritized (125–175 ms and 150–200 ms, respectively) processing of happy faces; (b) an overactivation of left‐hemispheric lobules (V/VI at 250–450 ms) for happy face processing at mid latencies; and (c) an over activation of midline (posterior vermis IX at 250–350 ms) cerebellar activity for angry face processing. All activation differences between the adolescents with ASD and TD controls were observed in the beta frequency band (mostly between ~20 and ~30 Hz) as originally hypothesized. Our findings partially confirm our hypothesis regarding the directionality of the cerebellar activations but provide new insights into the functional selectivity of the human cerebellum and a description of its temporal profile in response to emotional faces. More importantly, they provide information about the underlying neuropathology in the emotional face processing of adolescences with ASD.

### 4.1 Overactivation of left‐hemispheric lobules in ASD in response to happy faces

We report an increased activation of left VI and V for adolescents with ASD, when compared to controls, in response to happy faces. There are reports of left VI activity increase in response to positive (happy and surprised) faces, when compared to neutral faces (Schraa‐Tam et al., 2012), as well as right VI activity for positive stimuli (Keren‐Happuch et al., 2014) [but see (Baumann & Mattingley, 2012; Keren‐Happuch et al., 2014; Moulton et al., 2011; Styliadis et al., 2015) for VI and negative stimuli/faces]. VI has been found to co‐activate with cerebral areas of the mirror neuron system (i.e., insula, amygdala, frontal lobe), and thus engage in emotional regulation and contribute to goal‐directed behavior required to observe and respond to another person's facial (negative) expressions (Schraa‐Tam et al., 2012). Our findings of VI and V overactivation for adolescents with ASD compared to TD controls can be attributed to enhanced affective responsiveness to happy faces. In turn, recognizing and relating outcomes with positive stimuli to the extent of driving behavioral changes may require a level of emotional control that is difficult for individuals with ASD to achieve. Therefore, VI and possibly V activities could be related to the process for the accurate perception of salient and emotional cues in ASD that drives the selection of appropriate behavioral responses.

Lobule VI is a relatively new addition to the salience network (Habas et al., 2009) (centered on the dorsal anterior cingulate and frontoinsular cortices connected with subcortical limbic structures), which is involved in detecting, interpreting, and filtering relevant autonomic and emotional information (Seeley et al., 2007). The increased responsiveness of VI and V to positive facial cues (depicting emotions of other people) for adolescents with ASD, in comparison to TD controls, may confer advantages, such as the promotion of a more flexible and adaptive social behavior. Also, VI and V overactivation could signify the requirement for more emotional and/or perceptual resources for adolescents with ASD to decode this type of facial emotion correctly (Lindner & Rosén, 2006). Given the regulatory role of VI, this enhancement can be regarded as a compensatory mechanism for adolescents with ASD to achieve a level of adequate performance at the given task. Happy facial expressions are the first to be accurately recognized in TD children (Gao & Maurer, 2010), and are more salient to young children than angry faces (Todd, Evans, Morris, Lewis, & Taylor, 2011). The relatively spared processing of happy facial expressions in children with ASD is attributed to their enhanced experience and familiarity with happy faces (Farran, Branson, & King, 2011). Considering the developmental course of one's ability to correctly recognize emotional cues and that regardless of diagnosis, individuals from 12 years through to adulthood become increasingly more able to recognize correctly facial stimuli than younger children (Grossman, Klin, Carter, & Volkmar, 2000); our high functioning ASD group can theoretically be similarly adept at recognizing basic emotional expressions. Furthermore, the adolescents with ASD who participated in this study had no significant differences for either accuracy in responses, or in response latency to happy faces when compared to controls. Our suggestion is in line with several studies showing a happy face advantage (Calvo & Nummenmaa, 2008; Juth, Lundqvist, Karlsson, & Öhman, 2005).

Although we have considered the difference in cerebellar activity between adolescents with ASD and controls as advantageous and suggested that it highlights the role of VI and V lobules for the processing of salient facial stimuli, another account is that people with ASD are generally insensitive to social reward derived from happy faces (Sepeta et al., 2012). This is important as, in a previous study on almost the same cohort (Leung et al., 2015) that focused on the cerebral responses, the right anterior cingulum and left insula (core regions of the salient network) were underactivated within similar time windows when VI and V overactivation takes place. The insula's underactivation to happy faces in adolescents with ASD was considered to be consistent with the aforementioned insensitivity (Leung et al., 2015) and thus to highlight the dysfunction of the mechanisms subserving happy face processing in ASD (Farran et al., 2011). The implication under this notion is that our previous speculation may not apply for V activity which may be linked to deficits in reward processing. Nevertheless, the right anterior cingulum responds selectively for happy faces (Fusar‐Poli et al., 2009), and shows typical activation to happy faces in ASD (Critchley et al., 2000). Thus, it is plausible that lobules VI and V may serve different facets (typical and atypical) for happy face processing. V and VI activations onset around N170 (Bentin et al., 1996), a face‐sensitive component with usually (but not always) a larger response in the right cerebral hemisphere. Adolescents with ASD were previously found to respond to affective (happy and angry) faces with consistently larger left‐lateralized activations in the temporal regions in comparison to TD controls at latencies around the N170 (Leung et al., 2015). Given previous evidence showing that the cerebral hemispheric asymmetry, found in structure, behavior, and function (Toga & Thompson, 2003), correlates with the degree of functional asymmetry of the cerebellum (Wang, Buckner, & Liu, 2013), it is plausible that the left‐lateralized cerebellar activation could be a reasonable finding in ASD. In essence, future studies should further determine whether cerebellar overactivation in response to happy faces contributes to deficits in understanding social reward in ASD, and investigate the information flow along cerebro‐cerebellar pathways between regions of social reward relevance and lobules VI and V.

### 4.2 Overactivation of midline cerebellum in ASD in response to angry faces

We report increased activation of vermal IX for adolescents with ASD, when compared to TD controls, in response to angry facial features. This is not surprising as vermal IX has been previously found to activate in response to pictures of anger and disgust (Baumann & Mattingley, 2012) in healthy adults. Lobule IX shares intracerebellar functional connections with Crus I, VIIIA, and the anterior cerebellum (Bernard et al., 2012), regions that are consistently abnormal in ASD (Duerden, Mak‐Fan, Taylor, & Roberts, 2012; Stoodley, 2014). IX is considered part of the default mode network (Buckner, Krienen, Castellanos, Diaz, & Yeo, 2011; Habas et al., 2009) and is connected to the temporoparietal junction, which is implicated in social cognition in TD individuals (Mars et al., 2012). In ASD, IX was found to be consistently underactivated during socially awkward situations (Pantelis, Byrge, Tyszka, Adolphs, & Kennedy, 2015). Lobule IX is activated during social paradigms, particularly during abstract mentalizing (Van Overwalle et al., 2014), but also in response to the conflict experienced when healthy individuals break with social norms (Klucharev, Hytönen, Rijpkema, Smidts, & Fernández, 2009) and thus may signal social conflict. Damasio et al (2000) proposed that the vermis coordinates emotional responses and is further involved in the learned adjustment of those responses in a social setting. In light of these previous findings, we suggest that the vermal IX can guide conforming behavior and contributes to goal‐directed behavior that requires an individual to observe and respond to another person's facial negative expressions (Schraa‐Tam et al., 2012). As such, the cerebellar overactivation in response to angry faces for our ASD group indicates not only a deficiency in anger recognition but also in the adjustment of one's behavior to match the angry facial cues depicting emotions of other people and thus avoid conflicts with social norms. We consider such conflicts to result from an impairment in recognizing angry facial features that interfere with the expected behavior in individuals with ASD. Consistent with this notion, individuals with ASD often have less developed emotional concepts, and thus struggle to recognize the social norms and context associated with anger (Ashwin, Chapman, Colle, & Baron‐Cohen, 2006; Bal et al., 2010; Rieffe et al., 2007). However, the adolescents with ASD who participated in this study did not differ in terms of either accuracy in responses, or in response latency to angry faces when compared to controls. Taking into consideration that goal‐directed behavior requires continuous performance monitoring (Montague, King‐Casas, & Cohen, 2006), learning a behavioral pattern and thus being successful in social interaction is a learned trait. Any deviation from successful behavioral patterns calls for adjustments of behavior, such as social conformity. This need may trigger a prediction error signal (Schultz, 2006). Our suggestion is in line with the well‐documented ability of cerebellum to predict aspects of upcoming motor behavior and process prediction errors (Schmahmann & Pandya, 1997). Nevertheless, the differences in activity between the two groups in response to happy and angry faces may have depended on the brief presentation of faces since individuals with ASD have difficulties in facial recognition when the stimuli are presented from 500 ms (Rump, Giovannelli, Minshew, & Strauss, 2009) to 2–3 s (Critchley et al., 2000; Pichon, de Gelder, & Grèzes, 2009). Here, the stimuli were presented only for 80 ms, which may have amplified this effect, but we cannot further assess the importance of such manipulations.

### 4.3 Time course of cerebellar activity in response to emotional faces

Early overactivation of VI and V lobule for the ASD group in response to happy faces lasted from 125 to 175 ms and 150 to 200 ms, respectively, in a time window associated with N170. This component indexes the underlying neural mechanisms that subserve early stage face processing and facial affect for healthy individuals (Batty & Taylor, 2003; Bentin et al., 1996; Hinojosa, Mercado, & Carretié, 2015). N170 is considered a promising biomarker for ASD (Jeste & Nelson, 2009) as it is associated with deficits, such as slower face processing speed (McPartland, Dawson, Webb, Panagiotides, & Carver, 2004). Yet, no significant between‐group differences in response latencies were reported for our study's participants. At 250 ms, VI overactivates again in response to happy faces as well as vermal IX in response to angry faces. The N250 is another well‐known face‐related component that is sensitive to individual recognition (Schweinberger, Huddy, & Burton, 2004). N170 and N250 reflect the earliest structural and evaluative processes, respectively, typically involved in facial emotion perception. We theorize that the early onset of VI and V activities are linked to the between‐group difference in the early perceptual process involving the structural encoding of facial features. According to the notion, the processing of happy faces is prioritized relative to angry faces. We suggest that the subsequent VI and V activities in response to happy faces as well as the vermal IX activity in response to angry faces highlight between‐group differences in facial affect recognition which here takes place almost immediately after the differences in the structural face encoding (Streit et al., 1999).

### 4.4 Increased beta power for ASD facial processing

Beta‐band neural activity has been related to the processing of emotional stimuli (Güntekin & Başar, 2010; Miskovic & Schmidt, 2010) and increases during the first 500 ms of viewing emotional stimuli (Güntekin & Başar, 2014). Considering the beta‐band association with top‐down control mechanisms (Buschman & Miller, 2007), the increased beta‐band activity in adolescents with ASD for the processing of happy and angry faces may reflect compensation of inherent attentional deficits. Individuals with ASD can be easily distracted by irrelevant details (Burack, 1994), and require higher levels of perceptual load to successfully filter these out (Remington, Swettenham, Campbell, & Coleman, 2009). While our ASD group displayed a significantly lower accuracy in their responses in comparison with the TD group, they did not exhibit differences for either angry or happy faces in specific. The lack of significant differences in response latencies between ASD and TD indicates the absence of attentional bias across emotions or groups in our study. We suggest the increased beta activity in our findings for both happy and angry faces may thus imply a wide attentional spotlight for these types of facial expressions. Some support to our suggestion can be found in the reduced beta phase synchronization in regions related to face perception and affective processing (the fusiform and insula, respectively) that was previously shown during emotional perception of angry faces in adolescents with ASD for an almost similar cohort (Leung et al., 2014). The reduction was suggested to reflect reduced long‐range communication in brain networks that contribute to social cognition.

### 4.5 Limitations and future direction

Brain asymmetry is a critical aspect of human brain organization (Toga & Thompson, 2003). Variation in its development is thought to contribute to various neuropathologies in humans, including ASD. Individuals with ASD show atypical patterns of both structural and functional asymmetries (Kleinhans, Müller, Cohen, & Courchesne, 2008; Postema et al., 2019). Nevertheless, cerebellar asymmetries in response to affective faces have not been documented yet. It would be interesting to establish whether aspects of emotional face processing might be differentially localized in the two cerebellar hemispheres. However, our experimental design is inappropriate for this investigation due to the low number of stimuli presented in each hemifield for each type of facial expression and the rapid presentation of stimuli that do not allow for any lateral gaze shifts. Though we did not use eye‐tracking while participants were completing the task, we did take into consideration the issue of differential fixation between ASD and controls when designing the task. Future studies may include modified experimental designs (e.g., increased number of stimuli per facial type for each hemifield presentation) that will allow examining possible lateralization effects. Also, more adolescents with ASD were excluded from analyses due to lack of cerebellum coverage compared to the TD group, though the difference was not significant. During the experiments, adolescents with ASD faced more difficulties maintaining an optimal head position inside the MEG for covering cerebellum. This resulted in a higher number of datasets being excluded from further analysis for participants with ASD compared to TD controls who were more cooperative. On another account, a small number of the adolescents with ASD were on medication that can increase cerebellar metabolism (Moulton, Elman, Becerra, Goldstein, & Borsook, 2014; Volkow et al., 1997). However, the participants were not on stimulants for the testing, as if they were taking stimulants, they stopped the day before the study. Our study is cross‐sectional, restricted to adolescents, and did not include children or adults. It is unknown whether our findings change with age, and if more severe autistic subgroups are likely to exhibit greater atypical cerebellar activity. Our clinical and TD control groups were not matched for IQ, with our participants with ASD showing significantly lower IQ scores relative to controls. Therefore, such variations could have contributed to our findings.

### 4.6 Conclusions

The present study demonstrates distinct spatiotemporal patterns of cerebellar activity during happy and angry face processing in adolescents with ASD. The time‐dependent evolution in milliseconds and spatial localization of cerebellar activity, observed here, highlights that adolescents with ASD do not integrate cerebellar regions into the processing of emotional facial stimuli in a fashion similar to that of TD controls. Our findings suggest that ASD‐related emotional face processing during adolescence relates to a progression of overactivation of both hemispheric and midline cerebellar lobules. Specifically, the ASD group exhibits more activation in the left cerebellar hemispheric lobules for the processing of happy faces at both early and mid‐latency time windows, as well as more midline activity than controls for angry face processing at mid latencies. Our results fit well with the concept of distinct lobular localization of emotional processing (Moulton et al., 2011; Styliadis et al., 2015) at specific time windows (Styliadis et al., 2015). The activity required to process these two types of emotional expressions in adolescents with ASD was generated within different cerebellar lobules and is thus dependent on the anatomy of the recruited areas (hemisphere vs. vermis). Thus, these results suggest that the functional selectivity demonstrated in this study has an anatomical basis.

## CONFLICT OF INTEREST

The authors declare no conflict of interest.

## Supporting information


**Appendix** **S1**: Supplementary Information

## Data Availability

Data are available upon request.
