# Supplementary material for: Atypical spatiotemporal activation of cerebellar lobules during emotional face processing in adolescents with autism
Source: Hum Brain Mapp. 2021 Feb 2;42(7):2099–114. doi: 10.1002/hbm.25349 (PMC8046060; doi:10.1002/hbm.25349)
Supplement: Supplementary file 1 — Appendix S1: Supplementary Information [file HBM-42-2099-s001.docx]

# Supplementary Material

**Supplementary Table 1.** MEG temporal and occipital sensors covering the cerebellum per participant for ASD and TD (controls) adolescents

|  | | **Sensor Labels** |  |
| --- | --- | --- | --- |
| **ASD** |  | |  |
| 1 | **MLT**:34,35,44;**MLO**:31,32,33,41,42,43;**MRT**:35,44;**MRO**:31,32,33,41,42,43;**MZO**:02 | |  |
| 2 | **MLT**:34,35,44;**MLO**:31,32,33,41,42,43;**MRT**:35,44;**MRO**:31,32,33,41,42,43;**MZO**:02 | |  |
| 3 | **MLT**:34,35,43,44;**MLO**:31,32,33,41,42,43;**MRT**:33,35,43,44;**MRO**:31,32,33,41,42,43;**MZO**:02 | |  |
| 4 | **MLT**:24,25,26,32,33,34,35,42,43,44;**MLO**:21,22,31,32,33,41,42,43;**MRT**:23,24,25,26,32,33,34,35,42,43,44;**MRO**:21,22,31,32,33,41,42,43;**MZO**:01,02 | |  |
| 5 | **MLT**:33,34,35,42,43,44;**MLO**:31,32,33,41,42,43;**MRT**:34,35,43,44;**MRO**:31,32,33,41,42,43;**MZO**:02 | |  |
| 6 | **MLT**:33,34,35,43,44;**MLO**:31,32,33,41,42,43;**MRT**:34,35,44;**MRO**:21,31,32,33,41,42,43;**MZO**:01,02 | |  |
| 7 | **MLT**:34,35,43,44;**MLO**:21,31,32,33,41,42,43;**MRT**:23,24,25,26,33,34,35,43,44;**MRO**:21,22,31,32,33,41,42,43;**MZO**:01,02 | |  |
| 8 | **MLT**:43,44;**MLO**:31,32,33,41,42,43;**MRT**:35,44;**MRO**:31,32,33,41,42,43;**MZO**:02 | |  |
| 9 | **MLT**:35,44;**MLO**:31,32,33,41,42,43;**MRT**:34,35;**MRO**:31,32,33,41,42,43;**MZO**:02 | |  |
| 10 | **MLT**:34,35,44;**MLO**:21,22,31,32,33,41,42,43;**MRT**:23,24,25,26,31,32,33,34,35,43,44;**MRO**:11,21,22,31,32,33,41,42,43;**MZO**:01,02 | |  |
| 11 | **MLT**:33,34,35,43,44;**MLO**:21,22,31,32,33,41,42,43;**MRT**:34,35,42,43,44;**MRO**: 31,32,33,41,42,43;**MZO**:02 | |  |
| 12 | **MLT**:34,35,43,44;**MLO**:21,31,32,33,41,42,43;**MRT**:33,34,43,44;**MRO**: 21,31,32,33,41,42,43;**MZO**:01,02 | |  |
| 13 | **MLT**:23,24,25,26,34,35,43,44;**MLO**:11,21,22,31,32,33,41,42,43;**MRT**:24,25,26,34,35,43,44;**MRO**: 11,21,31,32,33,41,42,43;**MZO**:01,02 | |  |
| 14 | **MLT**:35,44;**MLO**:31,32,33,41,42,43;**MRT**:34,35,43,44;**MRO**:31,32,33,41,42,43;**MZO**:02 | |  |
| 15 | **MLT**:24,25,26,33,34,35,43,44;**MLO**:22,31,32,33,41,42,43;**MRT**:33,34,43,44;**MRO**:31,32,33,41,42,43;**MZO**:02 | |  |
| 16 | **MLT**:35,44;**MLO**:31,32,33,41,42,43;**MRT**:34,35,43,44;**MRO**:31,32,33,41,42,43;**MZO**:02 | |  |
| 17 | **MLT**:33,34,35,43,44;**MLO**:31,32,33,41,42,43;**MRT**:34,35,43,44;**MRO**:31,32,33,41,42,43;**MZO**:02 | |  |
| 18 | **MLT**:34,35,43,44;**MLO**:21,22,31,32,33,41,42,43;**MRT**:34,35,43,44;**MRO**:21,22,31,32,33,41,42,43;**MZO**:01,02 | |  |
| 19 | **MLT**:24,25,26,33,34,35,41,43,44;**MLO**:11,21,22,31,32,33,41,42,43;**MRT**:34,35,43,44;**MRO**:31,32,33,41,42,43;**MZO**:02 | |  |
|  |  | |  |
| **TD** |  |  |  |
| 1 | **MLT**: 34,35,43,44;**MLO**:31,32,33,41,42,43;**MRT**:33,34,35,43,44;**MRO**:31,32,33,41,42,43;**MZO**:02 | |  |
| 2 | **MLT**: 33,34,35,43,44;**MLO**:31,32,33,41,42,43;**MRT**:33,34,35,43,44;**MRO**:31,32,33,41,42,43;**MZO**:02 | |  |
| 3 | **MLT**: 33,34,35,43,44;**MLO**:31,32,33,41,42,43;**MRT**:33,34,43,44;**MRO**:31,32,33,41,42,43;**MZO**:01,02 | |  |
| 4 | **MLT**: 33,34,35,43,44;**MLO**:31,32,33,41,42,43;**MRT**:33,34,43,44;**MRO**:31,32,33,41,42,43;**MZO**:01,02 | |  |
| 5 | **MLT**: 33,34,35,43,44;**MLO**:31,32,33,41,42,43;**MRT**:,34,35;**MRO**:31,32,33,41,42,43;**MZO**:01,02 | |  |
| 6 | **MLO**:21,31,32,33,41,42,43;**MRT**:34,35,43,44;**MRO**:21,31,32,33,41,42,43;**MZO**:01,02 | |  |
| 7 | **MLT**: 34,35,43,44;**MLO**:31,32,33,41,42,43;**MRT**:34,35,43,44;**MRO**:31,32,33,41,42,43;**MZO**:02 | |  |
| 8 | **MLT**:24,25,26,33,34,35,43,44;**MLO**:11,12,21,31,32,33,41,42,43;**MRT**:24,25,26,33,34,35,43,44;**MRO**:11,12,21,22,.31,32,33,41,42,43;**MZO**:01,02 | |  |
| 9 | **MLT**:24,25,26,33,34,35,43,44;**MLO**:21,22,31,32,33,41,42,43;**MRT**:24,25,26,34,35,43,44;**MRO**:21,22,31,32,33,41,42,43;**MZO**:01,02 | |  |
| 10 | **MLT**: 34,35,43,44;**MLO**:31,32,33,41,42,43;**MRT**:34,35,43,44;**MRO**:31,32,33,41,42,43;**MZO**:02 | |  |
| 11 | **MLT**: 34,35,43,44;**MLO**:31,32,33,41,42,43;**MRT**:34,35,43,44;**MRO**:31,32,33,41,42,43 | |  |
| 12 | **MLT**: 34,35,43,44;**MLO**:31,32,33,41,42,43;**MRT**:34,35,43,44;**MRO**:31,32,33,41,42,43;**MZO**:01,02 | |  |
| 13 | **MLT**:33,34,35,43,44;**MLO**:31,32,33,41,42,43;**MRT**:33,34,35,43,44;**MRO**:31,32,33,41,42,43;**MZO**:02,02 | |  |
| 14 | **MLT**:24,25,26,34,35,43,44;**MLO**:21,22,31,32,33,41,42,43;**MRT**:24,25,26,34,35,43,44;**MRO**:21,22,31,32,33,41,42,43;**MZO**:01,02 | |  |
| 15 | **MLT**:24,25,26,34,35,43,44;**MLO**:11,21,22,31,32,33,41,42,43;**MRT**:34,35,43,44;**MRO**:21,22;**MZO**:01,02 | |  |
| 16 | **MLT**: 34,35,43,44;**MLO**:31,32,33,41,42,43;**MRT**:34,35,43,44;**MRO**:31,32,33,41,42,43;**MZO**:02 | |  |
| 17 | **MLT**:24,25,26,33,34,35,43,44;**MLO**:21,22,31,32,33,41,42,43;**MRT**:24,25,26,34,35,43,44;**MRO**:21,22,31,32,33,41,42,43;**MZO**:01,02 | |  |
| 18 | **MLT**:24,25,26,33,34,35,43,44;**MLO**:21,22,31,32,33,41,42,43;**MRT**:24,25,26,33,34,35,43,44;**MRO**:21,22,31,32,33,41,42,43;**MZO**:01,02 | |  |
| 19 | **MLT**:13,14,15,16,24,25,26,33,34,35,43,44;**MLO**:11,12,21,22,31,32,33,41,42,43;**MRT**:13,14,15,16,24,25,26,34,35,43,44;**MRO**:11,12,21,22,31,32,33,41,42,43;**MZO**:01,02 | |  |
| 20 | **MLT**:23,24,25,26,33,34,35,43,44;**MLO**:21,22,31,32,33,41,42,43;**MRT**:23,24,25,26,34,35,43,44;**MRO**: 21,22,31,32,33,41,42,43;**MZO**:01,02 | |  |
| 21 | **MLT**:24,25,26,33,34,35,43,44;**MLO**:21,22,31,32,33,41,42,43;**MRT**: 24,25,26,33,34,35,43,44;**MRO**: 21,22,31,32,33,41,42,43;**MZO**:01,02 | |  |
| 22 | **MLT**:24,25,26,33,34,35,43,44;**MLO**:21,22,31,32,33,41,42,43;**MRT**: 23, 24,25,26,33,34,35,43,44;**MRO**: 21,22,31,32,33,41,42,43;**MZO**:01,02 | |  |
| 23 | **MLT**:34,35,43,44;**MLO**:31,32,33,41,42,43;**MRT**:33,34,35,43,44;**MRO**:31,32,33,41,42,43;**MZO**:01,02 | |  |
